# Prognosis in Burns and Scalds

**Published:** 1907-08-24

**Authors:** A. G. L. Reade, J. W. Scott Macfie

**Affiliations:** House Physician and late Senior House Surgeon, the Radcliffe Infirmary, Oxford; Assistant House Surgeon, The Radcliffe Infirmary, Oxford


### Resident Medical Officers' Department.

[Contributions to this Column are invited, and, if accepted, will be paid for.]

### PROGNOSIS IN BURNS AND SCALDS.

By A. G. L. Reade, M.R.C.S.Eng., L.R.C.P.Lond., House Physician and late Senior House Surgeon, the Radcliffe Infirmary, Oxford; and J. W. Scott Macfie, B.A.Cantab., M.B., Ch.B.Edin., Assistant House Surgeon, The Radcliffe Infirmary, Oxford.

Of all the cases with which the casualty officer is called upon to deal perhaps none require his serious consideration so much as those injuries arising from slight burns and scalds. It is often difficult to realise the danger of a small burn or scald, and the house surgeon may be inclined to grudge a bed to a case that might, it would seem, be met by a simple dressing. And yet, if he should yield to the pressure of an overcrowded ward and allow such a case to go out, he may subsequently have reason to reproach himself for the most serious consequences. Briefly, the prognosis in cases of injury from burns and scalds may be said to depend on five things, namely: The age of the patient, the extent of the injury, its site, its depth, and the subsequent occurrence or avoidance of sepsis. A burn is always serious at either extreme of life, and should be treated with corresponding care. In children, in addition, the relative area involved is naturally greater, and this is a further danger. When a third of the body is involved, death from shock and toxæmia is invariably the result, but, as is illustrated in one of our cases detailed below, death may result from even a very small scald. In some situations burns are very much more dangerous than in others. This is especially the case in burns about the head and trunk, which must always be looked upon as serious. With sepsis, which, of course, prejudices prognosis, whenever it occurs, may be associated the other secondary complications which occur in cases of burns and scalds—such as lung troubles, the contraction of cicatricial tissue, œdema glottidis, and the occurrence of duodenal ulcer, which, although always described among the other sequelæ of burns, is very rarely seen in actual experience.

Three cases which have recently come under our notice illustrate admirably the importance of regarding even slight burns and scalds as serious injuries.

Case 1.—The first case is that of a child two years of age—a little girl who accidentally sat down in a basin of very hot water, and was brought up to the hospital immediately after the accident. On examination she was found to have a red and inflamed area over one buttock; there was no desquamation, no destruction of the skin, but simply an erythematous area. The injury was regarded as trivial, but as a precautionary measure the child was admitted to one of the wards. For two days the patient appeared to be doing well, but on the third day cyanosis set in, her pulse became shallow and rapid, and in the evening, despite all efforts to improve her condition, she died. At the post-mortem examination it was found that she had acute congestion of both lungs, which had no doubt been the immediate cause of death.

Case 2.—The second case is that of a laundry maid, who had the misfortune to get her hand burnt by the hot rollers used in her work. She appeared to be suffering from a burn of the second degree, involving the palmar aspect of all the digits, and complained of great pain. She was admitted to the hospital, and treated in the first instance with picric acid. Later she was treated daily for some time with two-hour boric baths, and oiled silk or boric ointment dressings. In a very few days the pain subsided completely, and the fingers were cautiously extended. But, when the dead skin began to slough away, and the wounds discharged some pus, it was thought necessary to readmit the patient, so that she might be treated by a continuous boric bath. In spite of this treatment the sloughing process extended so deeply that the phalanges were exposed in the third, fourth and fifth digits, and the palmar aspect of the index finger was reduced to a mass of granulation tissue.

Case 3.—The third case is typical of a lamentably common accident. It is that of a little boy, three years of age, who had sucked the spout of a kettle of hot water. On admission the child was not dyspnœic, but, as was anticipated, œdema glottidis soon succeeded, so that the same evening it became necessary to perform tracheotomy to relieve the dyspnœa and cyanosis. On the third day after admission the boy died. At the post-mortem examination congestion of the lungs was found in addition to the œdema of the glottis. When a child is brought in with a history of having sucked a kettle spout, it should not be allowed to leave the hospital for some days, even although it presents no alarming symptoms at the outset.

These three cases illustrate some of the difficulties that are encountered in estimating the prognosis in cases of injuries from burns or scalds, which, on superficial examination, appear to be inconsiderable. If the little girl in the first case had been allowed to leave the hospital and had subsequently died at home, it would, we think, have been difficult for the house surgeon to justify before the jury of the coroner's court his action in discharging the case. And in the second case, if a favourable prognosis had been given on the appearances at first presented, the surgeon could hardly have been surprised if his patient had suspected incompetent treatment, when later on he was compelled to confess that her injuries were so serious as to necessitate amputation of the fingers perhaps, or at best a laborious course of skin grafting. And therefore we would urge that in considering the injuries arising from slight burns or scalds a very guarded prognosis should be given both as to the immediate and the ultimate consequences.

